# National Confederation of State Medical Examining and Licensing Boards (*Abstract of Proceedings*)

**Published:** 1909-08

**Authors:** Murray Galt Motter


### National Confederation of State Medical Examining and Licensing Boards

(*Abstract of Proceedings.*)

*Held at Atlantic City, N. J., June 7, 1909.*

Reported by MURRAY GALT MOTTER, M. D., Secretary.

THE Nineteenth Annual Convention of the National Confederation of State Medical Examining and Licensing Boards, was called to order by the Vice-President, Dr. A. Ravogli, of Ohio, at Hotel Marlborough, Atlantic City, N. J., at 10.15 A. M. There were present Drs. Edwin B. Harvey and W. T. Councilman, Massachusetts; L. F. Bennett, Wisconsin; Charles A. Tuttle, Connecticut; F. C. Zapffe and A. D. Bevan, Illinois; W. J. Means, N. R. Coleman, J. A. Duncan, R. A. Baker and M. A. Jerome, Ohio; Abraham Flexner, of the Carnegie Foundation; N. P. Colwell, of the Council on Medical Education; Henry Beates, J. C. Guernsey, Augustus Korndoerfer, Edward Cranch, C. S. Middleton, H. L. Northrop, and D. P. Maddux, Pennsylvania; A. B. Briggs, G. P. Swarts and J. H. Bennett, Rhode Island; E. L. B. Godfrey, New Jersey; W. W. Potter, W. S. Ely, A. Vander Veer, R. H. Williams, and A. T. Bristow, New York; F. S. Raymond, Tennessee; George Dock, Tulane University; W. H. Welch, Maryland; and S. D. Van Meter, Colorado.

The minutes of the eighteenth annual convention, held in Chicago, June 1, 1908, were read and approved.

After appointing Dr. Harvey and Dr. Guernsey as an auditing committee, Dr. Ravogli called Dr. Guernsey to the chair and delivered his address, which consisted of a eulogy of the late Dr. Thomas J. Happel, and a paper on The Necessity of a Practical Test for Candidates in the State Examinations.

On motion, a committee was appointed to draft resolutions commemorating Dr. Happel, and a recess of five minutes was taken out of respect to Dr. Happel. The following resolutions were submitted by the Committee and adopted by the Confederation:

WHEREAS, By the death of Dr. Thomas J. Happel, of Trenton, Tennessee which occurred on the 24th day of May last, the National Confederation of State Medical Examining and Licensing Boards suffers the loss of its honored President, therefore,

*Resolved,* That in the death of Doctor Happel the medical profession has lost one of its most valued members and the community an honored citizen. Doctor Happel spent the greater part of his active life in the upbuilding of medical education and the medical profession in this country.

*Resolved,* That this Confederation extends its most profound sympathy to the family and relatives of the late Doctor Happel.

*Resolved,* That a copy of these resolutions, duly engrossed, be forwarded to the family of Doctor Happel, and the same be spread upon the records of this Confederation.

Atlantic City, N. J., June 7, 1909.

The report of the executive council was read by the chairman, Dr. N. R. Coleman. Acting upon the recommendations contained in this report, a committee of five, all of whom are members of state boards, was appointed to investigate and report at the succeeding meeting of the Confederation on the subject of clinical instruction in medical colleges; and provision was made for the presentation of this subject by clinical teachers themselves. The secretaries of each of the state and territorial examining boards, and of the District of Columbia, were appointed as a Committee of Official Correspondents of the Confederation.

Dr. N. P. Colwell then read a paper on The Need, Methods and Value of Medical College Inspection, following which the report on Standing of Medical Colleges was read by Dr. James

A. Duncan. These papers were discussed by Drs. Abraham Flexner, of the Carnegie Foundation; W. J. Means, R. A. Baker, Henry Beates, M. G. Motter, N. R. Coleman; Edwin B. Harvey, and F. C. Zapffe.

After a recess for luncheon, Dr. J. C. Guernsey read the report of the Examinations Committee. This report was referred to the executive council for further consideration, and on the basis of recommendation contained therein the following resolution was adopted:

WHEREAS, A mixed examination, oral, practical and written, of applicants for medical licensure in every state is important, therefore be it

*Resolved,* That this Confederation earnestly recommends to the various state boards now restricted as to their methods of conducting examinations to take proper steps to secure amendatory legislation to enable them so to conduct their examinations.

WHEREAS, It is generally recognised that the time available for instruction in therapeutics, materia medica and pharmacology does not suffice for the study of all the known drugs; and

WHEREAS, It is also recognised that the limited time can be devoted to best advantage, to acquiring a thorough familiarity with the characters, actions and uses of the important drugs at the expense of those of doubtful or negative value; therefore be it

*Resolved,* That the National Confederation of State Medical Examining and Licensing Boards urges upon its constituents the advisability of confining examination questions to the most important preparations of the more important drugs.

Under the head of Symposium on Examinations, the following papers were presented: Practical versus Theoretical Examinations, by Dr. W. T. Councilman; The Inadequacy of the Written Examination, by Dr. Edwin B. Harvey; What Branches or Portions of Branches Should be Included in the Written, and What in the Oral Examinations? by Dr. J. C. Guernsey; Is It to the Best Interests of Students and Medical Examining Boards to have Divided Examinations? by Dr. William Warren Potter; the Feasibility of Practical Examinations, by Dr. Augustus Korndoerfer; How Can Practical Examinations be Graded and Recorded? by Dr. L. F. Bennett.

Dr. M. G. Motter, as a part of the secretary's annual report, reviewed briefly the work of the sub-committee on the Teaching of Materia Medica and Therapeutics of the Council on Medical Education, A. M. A. The recommendations contained in this report were referred to the executive council, and the following resolutions were finally adopted:

*Resolved,* That it recommends to their attention the report of the Sub-Committee on the Teaching of Materia Medica, and the like, of the American Medical Association's Council on Medical Education; and be it further

*Resolved,* That the President of the National Confederation of State Medical Examining and Licensing Boards be, and he is, hereby authorised and instructed to appoint a committee of three to confer with the council on medical education and to compile and report to this Confederation a list of the more important drugs and their preparations to which the examinations of the constituent boards may be confined, this list to be published in the *Journal of the American Medical Association* as with the approval and endorsement of the Confederation.

The several papers in this symposium on examinations were discussed by Drs. Dock, Northrop, Beates, Middleton, Van Meter, Vander Veer, Motter, Swarts, Harvey, Councilman, Korndoerfer, Bennett and Guernsey.

The auditing committee reported that the accounts of the secretary-treasurer had been examined and found correct.

Dr. Edward Cranch, of Erie, Pa., Dr. George MacDonald, of Washington, D. C., and Dr. A. B. Briggs, of Providence, R. I., were elected to membership in the Confederation.

The secretary and the chairman of the executive council were authorised to make the necessary arrangements for the Convention of 1910.

The following officers and members of the executive council were elected for the ensuing year: president, Dr. A. Ravogli, Ohio; vice-president, Dr. J. C. Guernsey, Pennsylvania; 2d vice-president, Dr. Charles A. Tuttle, Connecticut; secretary-treasurer, Dr. M. G. Motter, Washington, D. C.

Executive Council—N. R. Coleman, chairman; Edwin B. Harvey, James A. Duncan Henry Beates, D. P. Maddux.

The following committees were appointed: Committee on Clinical Instruction—Henry Beates, chairman; Charles A. Tuttle, Fred C. Zapffe, M. J. Lewi, L. F. Bennett.

Committee to compile list of drugs—M. G. Motter, chairman; J. C. Guernsey, George MacDonald.

At 10.30 P. M., the Confederation adjourned to meet on the day preceding the annual convention of the American Medical Association in 1910 and at the place of that convention.

1841 Summit Place, Washington, D. C.

---

In persons who have had malaria any operation which taxes the vital powers may provoke a recurrence, and it is important to differentiate the resulting fever from that due to a septic process.—*International Journal Surgery.*

